# Serum levels of free light chains and syndecan-1 in patients with rheumatoid arthritis and systemic lupus erythematosus

**DOI:** 10.1093/rheumatology/keae623

**Published:** 2024-11-07

**Authors:** Valeria Carnazzo, Francesca Gulli, Valerio Basile, Riccardo Di Santo, Benedetta Niccolini, Serena Redi, Ilaria Vinante, Cecilia Napodano, Krizia Pocino, Gian Ludovico Rapaccini, Marco Maria Lizzio, Mariapaola Marino, Gabriele Ciasca, Umberto Basile

**Affiliations:** Unità Operativa Complessa di Patologia Clinica, Ospedale Santa Maria Goretti, A.U.S.L., Latina, Italy; Clinical Biochemistry Laboratory, I.R.C.C.S. ‘Bambino Gesu’ Children’s Hospital, Rome, Italy; Clinical Pathology Unit and Cancer Biobank, Department of Research and Advanced Technologies, IRCCS Regina Elena National Cancer Institute, Rome, Italy; Dipartimento di Neuroscienze, Sezione di Fisica, Università Cattolica del Sacro Cuore, Fondazione Policlinico Universitario ‘A. Gemelli’ I.R.C.C.S., Rome, Italy; Dipartimento di Scienze della Vita, della Salute e delle Professioni Sanitarie, Link Campus University, Rome, Italy; Istituto di Fotonica e Nanotecnologie, Consiglio Nazionale delle Ricerche IFN-CNR, Rome, Italy; Dipartimento di Neuroscienze, Sezione di Fisica, Università Cattolica del Sacro Cuore, Fondazione Policlinico Universitario ‘A. Gemelli’ I.R.C.C.S., Rome, Italy; Unità Operativa Complessa di Patologia Clinica, Ospedale Santa Maria Goretti, A.U.S.L., Latina, Italy; Unità Operativa Complessa di Patologia Clinica, Ospedale Santa Maria Goretti, A.U.S.L., Latina, Italy; Department of Laboratory of Medicine and Pathology, S. Agostino Estense Hospital, Modena, Italy; Unità Operativa Complessa di Patologia Clinica, Ospedale Generale di Zona San Pietro Fatebenefratelli, Rome, Italy; Dipartimento di Medicina e Chirurgia Traslazionale, Università Cattolica del Sacro Cuore, Fondazione Policlinico Universitario ‘A. Gemelli’ I.R.C.C.S., Rome, Italy; UOC di Reumatologia, Fondazione Policlinico Universitario ‘A. Gemelli’ I.R.C.C.S., Rome, Italy; Dipartimento di Medicina e Chirurgia Traslazionale, Università Cattolica del Sacro Cuore, Fondazione Policlinico Universitario ‘A. Gemelli’ I.R.C.C.S., Rome, Italy; Dipartimento di Neuroscienze, Sezione di Fisica, Università Cattolica del Sacro Cuore, Fondazione Policlinico Universitario ‘A. Gemelli’ I.R.C.C.S., Rome, Italy; Unità Operativa Complessa di Patologia Clinica, Ospedale Santa Maria Goretti, A.U.S.L., Latina, Italy

**Keywords:** biomarkers, SDC-1, FLC, autoimmunity, SARD, RA, SLE

## Abstract

**Objectives:**

Systemic autoimmune rheumatic diseases (SARDs) are characterized by chronic inflammation. Reliable biomarkers are crucial for diagnosis, monitoring disease progression and therapeutic responses. This study explores serum syndecan-1 (SDC-1) as a biomarker for these conditions and its relationship with free light chain (FLC) levels.

**Methods:**

A retrospective analysis was performed on sera from 60 patients with rheumatoid arthritis (RA) and from 60 with systemic lupus erythematosus (SLE), alongside 50 healthy donors (HD). Κ- and λ- FLCs were determined by turbidimetric assay, while SDC-1 levels were determined by ELISA. Kruskal–Wallis test, Wilcoxon Mann–Whitney *U* test, multivariable linear regression and Spearman’s correlation were employed to compare biomarker levels across groups and to explore correlations.

**Results:**

SDC-1, κ-FLC and λ-FLC were significantly increased in RA and SLE patients compared with HD (*P* < 0.001), while no significant differences in the κ/λ ratio were observed among the groups (*P* = 0.4). A significant difference in subject age was also identified. However, multivariate regression analysis indicated that RA and SLE are significantly associated with the levels of these markers, with minimal confounding by age. A significant correlation was observed separately in all groups between the FLC markers. Conversely, no correlation was detected between SDC-1 and FLCs, nor between these markers and age or disease activity indices.

**Conclusion:**

Elevated serum levels of FLCs and SDC-1 in RA and SLE patients compared with HD underscore their potential as biomarkers for SARDs. The findings also suggest sustained plasma cell activation, supporting the multifaceted role of SDC-1 in the pathogenesis of SARDs.

Rheumatology key messages

- Serum levels of FLCs and SDC-1 were studied for the first time together in SARDs after discontinuing immunosuppressants.
- SDC-1, κ-FLC and λ-FLC levels significantly increased in RA and SLE patients compared to HD; no significant differences in κ/λ ratio among groups.
- SDC-1 and FLC levels increase independently of disease activity in SLE and RA, highlighting their multifaceted role in immune response modulation.

## Introduction

Systemic autoimmune rheumatic diseases (SARDs) represent a spectrum of life-long, debilitating disorders. Among SARDs, systemic lupus erythematosus (SLE) and rheumatoid arthritis (RA) lead to increased morbidity and mortality due to multi-organ involvement and impose a significant healthcare burden for the costs associated with their management. The handling of these diseases is complicated by their unpredictable relapsing-remitting or flare patterns, which lack reliable predictors for frequency or severity. In this context, measuring levels of autoantibodies (auto-Abs), the primary hallmark of autoimmunity, serves as a critical diagnostic tool [1–3].

Despite the assessment of autoantibodies, in many cases the diagnosis may remain puzzling and other serological biomarkers are widely desired and currently investigated such as, among others, free light chains (FLCs) of immunoglobulins [4–12]. Produced in excess of heavy chains during immunoglobulin synthesis by plasma cells, FLCs serve not only as direct biomarkers of B-cell activation but also play a suggested role in the pathogenesis and progression of the disease [13–15]. Serum levels of FLCs have been found to correlate with disease activity indices in SLE patients [14]. Similarly, Deng *et al.* demonstrated that elevated serum FLC levels precede the development of RA and could be useful for monitoring B-cell activity and disease progression [13]. A comparison of FLC levels in RA, SLE and other SARDs was made by Gulli *et al.*, suggesting their potential for identifying more aggressive forms [16].

**Figure 1.**
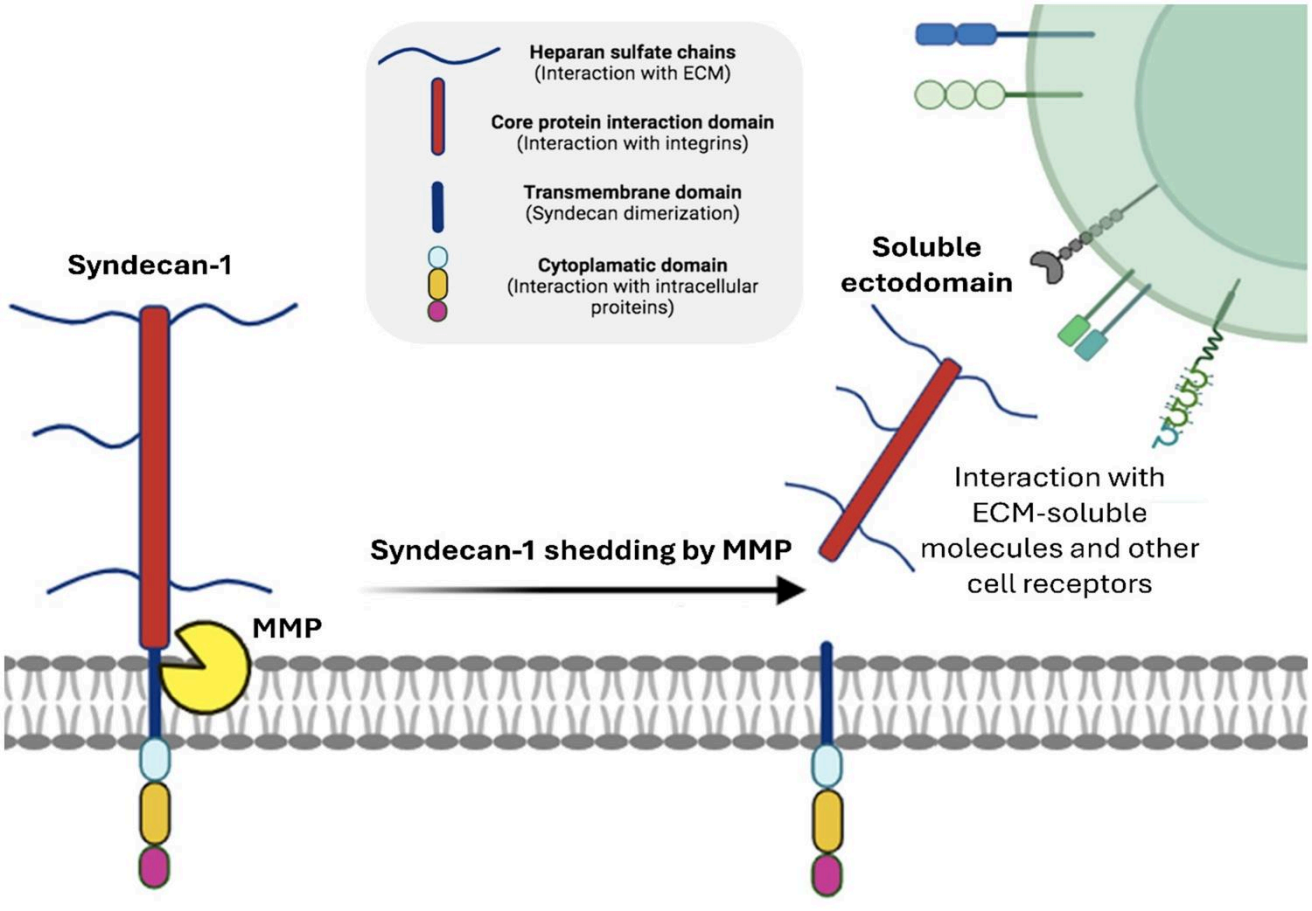
Structure and shedding mechanism of syndecan-1. Syndecan-1 is composed of three main domains: the extracellular domain (ectodomain), which consists of a core protein interaction domain and heparan sulphate chains that interact with growth factors and fibronectin; a single transmembrane domain that drives syndecan dimer formation; and the highly conserved cytoplasmic domain, composed of three sub-domains that allow interaction with intracellular proteins. The shedding process involves the proteolytic cleavage of the syndecan-1 ectodomain near the plasma membrane by enzymes such as metalloproteinases (MMPs). Heparan sulphate (HS) chains can be further cleaved by heparanase. Soluble syndecan-1 is in dynamic interplay with the extracellular matrix (ECM), cytokines, chemokines, several receptors and various growth factors, acting as co-receptors and mediating numerous signalling pathways

Emerging research has begun to highlight other biomarkers with significant implications for autoimmunity, including syndecan-1 (SDC-1) [17–19]. Also known as CD138, SDC-1 is a transmembrane heparan sulphate proteoglycan ubiquitously found on the cell surface and nuclear membrane (Fig. 1) [20]. It plays a pivotal role in cell adhesion, migration and signalling, which are crucial for maintaining tissue integrity and determining cellular behavior. Its regulation is mediated through proteolytic cleavage by enzymes such as heparanase, which release its bioactive extracellular domain into the circulation, thereby amplifying its role as a cellular modulator (Fig. 1). The release of soluble SDC-1 into the circulation and its accumulation in various tissues can alter the expression and availability of growth factors and signalling molecules, influencing the tissue microenvironment [21–24]. Actively shed by most myeloma cells, it has been suggested for monitoring and follow-up of patients with plasma cell proliferative disorders and explored, in such settings, in parallel with FLCs [25].

Intriguingly, dysregulation of SDC-1 has also been suggested to play a direct role or act as a trigger in autoimmune diseases [26–33]. Systemic inflammation leads to its cleavage and subsequent release into the circulation, a phenomenon particularly noted in SLE, especially among patients with lupus nephritis. Kim and colleagues have proposed that residual SDC-1 molecules could activate quiescent autoreactive plasma cells to secrete autoantibodies, potentially contributing to the development of SLE and its flares [28]. Furthermore, anti-rheumatic treatments in RA have been associated with reductions in serum SDC-1 levels, reflecting a decreased shedding from the endothelial glycocalyx [30]. These observations suggest that SDC-1 levels could also serve as indicators of therapeutic response in RA and SLE, enhancing management of these conditions.

To the best of our knowledge, FLCs and SDC-1 levels have never been simultaneously investigated in the same group of SARD patients. In this study, we examined their levels in RA and SLE patients to evaluate their potential as disease biomarkers and their relation to disease activity.

## Patients and methods

### Patients

We retrospectively analysed sera from 120 patients (60 with RA, 60 with SLE) enrolled by the Dipartimento di Medicina Interna e Gastroenterologia, Fondazione Policlinico Universitario ‘A. Gemelli’ I.R.C.C.S. (Rome, Italy). The study included patients with RA or SLE who, after a period of active treatment, were in low-to-moderate disease activity on a stable hydroxycloquine regimen for 3–6 months. None of the participants underwent plasmapheresis or received high-dose intravenous immunoglobulins during the study. Fifty healthy donors (HD) were included as controls. All participants included in this study were of Caucasian descent.

The inclusion criteria for RA patients were detection of antibodies against cyclic citrullinated peptide (anti-CCP) at moderate or high levels and positivity for rheumatoid factor (RF). For SLE patients, the criteria included antinuclear antibodies (ANA) positivity at titres ≥1:640 with a homogeneous pattern, positive results from an immunofluorescence assay for antibodies to native dsDNA using the kinetoplast of *Crithidia luciliae* as a substrate, and measurements from an extractable nuclear antigen (ENA) panel.

Subjects with diagnoses of plasma cell dyscrasia disorders, chronic diseases, cancer and renal failure were excluded from the study, as were female patients who were pregnant or breastfeeding in the last six months. Demographic (age and sex), clinical and laboratory parameters were collected retrospectively. All the enrolled patients fulfilled the latest versions of the classification criteria for RA and SLE [34, 35]. Blood and urine samples were collected between January 2010 and December 2016. Common biochemical parameters, including urea, creatinine, uric acid, serum lipids, electrolytes, albumin, haemoglobin and proteinuria were measured at baseline, according to standard methods in the routine clinical laboratory. All patients had an estimated glomerular filtration rate (eGFR) ≥60 mL/min/1.73 m^2^, suggesting the absence of significant renal impairment. Routine laboratory assays, including blood urea nitrogen (BUN), serum creatinine assessment, and urinalysis for research of protein, red blood cells and cellular casts confirmed the absence of renal disease. Moreover we measured a normal creatinine clearance and a normal protein excretion in 24-h urine collections in all patients and controls (normal creatinine excretion is 1000 mg/24 h/1.75 m^2^; normal protein excretion is 150–200 mg/24 h/1.75 m^2^; normal urinary protein-to-creatinine ratio is <0.2).

### Laboratory investigation

Sera were collected and stored at −80°C. All samples were tested simultaneously. Rheumatoid factor (RF) measurements were conducted according to the manufacturer’s protocol using the BNII analyser from Siemens Healthcare, Erlangen, Germany. An RF value >20 IU/ml was considered positive. Anti-CCP IgG evaluation was performed using a chemiluminescence immunoassay (CLIA) with QUANTA Flash^®^ CCP3 (Inova, Werfen, San Diego, CA, USA). Levels of anti-CCP IgG are considered negative with results <20 units, weak with 20–40 units, moderate with 40–60 units and high with >60 units; serum levels are reported in U/ml, according to the manufacturer. ANA titres were determined by IIFA using a HEp-2 cell line. ANA were considered positive at titres ≥1:80. The detection of ANA with a homogeneous pattern by IIFA was followed by a synthetic double-stranded DNA (dsDNA) chemiluminescent immunoassay, with serum levels reported in U/ml (9.8–35 negative; 35–45 uncertain; >45 positive), according to the manufacturer’s instructions. Positive specimens for anti-dsDNA using a CLIA with QUANTA Flash^®^ (Inova, Werfen, San Diego, CA, USA) were further evaluated for antibodies to native dsDNA by an immunofluorescence test using the kinetoplast of *Crithidia luciliae* as a substrate. This confirmation method has been assessed and demonstrated high specificity as a diagnostic test for SLE. FLCs were assessed using a turbidimetric assay (Freelite Human Kappa and Lambda Free Kits, The Binding Site, Birmingham, UK) performed with the Optilite^®^ instrument (The Binding Site; free κ normal range: 3.3–19.4 mg/l; free λ normal range: 5.7–26.3 mg/l). A κ/λ ratio <0.26 or >1.65 was considered abnormal, according to the manufacturer; calibrators and controls were diluted to the appropriate concentrations for serum determinations, following the manufacturer’s instructions.

Serum SDC-1 levels were determined using ELISA kits specific for human SDC-1 (Diaclone Research, Besançon, France). The assay was performed according to the manufacturer, as follows: 100 ml of serum were added to pre-coated wells and incubated with anti-SDC-1 biotinylated antibody. The wells were washed and horseradish peroxidase-streptavidin conjugate was added. After another wash, the substrate was added, and the reaction was stopped after 20 min. The absorbance was then read at 450 nm. Serum concentrations of SDC-1 were expressed in ng/ml. The operator was blinded to the clinical information of the samples.

### Ethics statement

The study was approved by the Università Cattolica of Rome Ethics Committee (Immuno-HVR; ID 2080) and conducted in accordance with the Helsinki Declaration (Seoul 2008 revision). All patients and HDs provided informed consent.

### Statistical analysis

Data analysis was conducted using R (version 4.2.1). Discrete variables were presented as absolute frequency/percentages. Continuous variables underwent normality testing using the Shapiro–Wilk test, highlighting deviations from normality. Consequently, continuous variables were represented as the median and interquartile range (Q3–Q1). Tabular data were reported using ‘gtsummary’ [36]. For comparisons, the χ^2^/Fisher’s exact test was employed for categorical variables and the Wilcoxon Mann–Whitney *U* test for continuous variables. Correlations were assessed using Spearman’s coefficient. Only significant correlations were reported and organized in correlation maps [37–39] (significance, *P* < 0.05).

## Results

**Figure 2.**
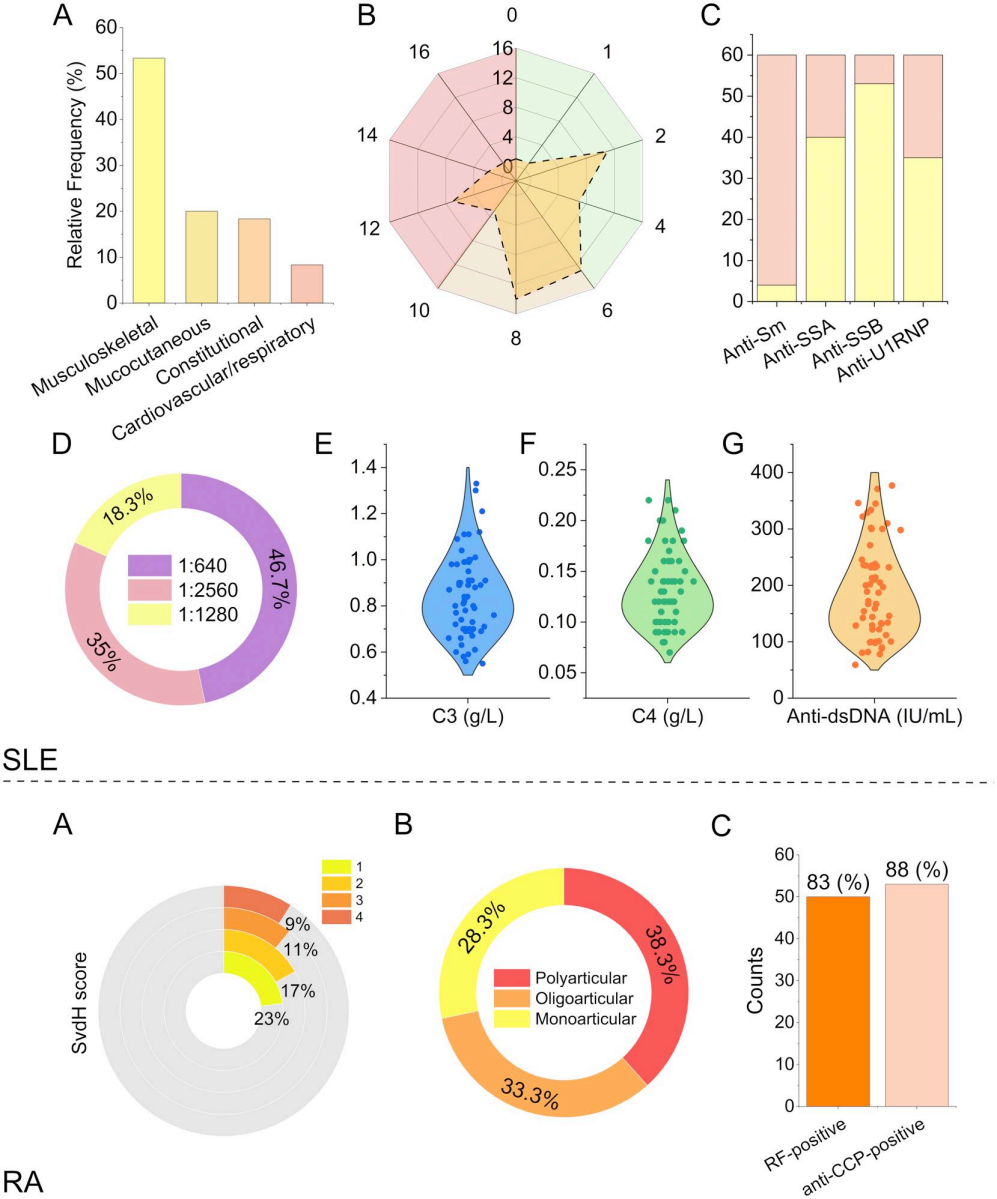
Visual representation of clinical and laboratory data for SLE and RA patients. (**A**) prevalence of musculoskeletal issues; (**B**) distribution of SLEDAI-2K disease activity scores; (**C**) counts of patients with positive extractable nuclear antigens (ENAs), highlighting Anti-SS-B/La antibodies; (**D**) breakdown of ANA titres; (**E**–**F**) levels of complement proteins C3 and C4; and (**G**) anti-dsDNA levels indicating autoimmune activity; (**H**) distribution of Sharp/van der Heijde (SvdH) scores depicting varying degrees of joint damage; (**I**) patterns of initial articular involvement; and (**J**) prevalence of rheumatoid factor (RF)

In Table 1, we outline the clinical and laboratory characteristics of patients diagnosed with SLE and RA. These data are further explored in Fig. 2 for SLE. Fig. 2A shows the pattern of organ involvement, highlighting a higher prevalence of musculoskeletal issues. Fig. 2B displays the distribution of SLEDAI-2K scores, showing that the majority of patients exhibit mild or moderate disease activity, with only a few subjects having a score of 12 or higher. Fig. 2C displays the counts of patients positive for ENAs, revealing a significant presence of Anti-SS-B/La antibodies, which indicates an active immune response. Fig. 2D details the ANA titres: 35% of patients exhibit antibodies at the highest dilution of 1:2560, suggesting a strong autoimmune response; 18.3% are positive at a lower dilution of 1:1280; and 46.7% test positive at the least concentrated dilution of 1:640, reflecting varied intensities of autoimmune activity. Fig. 2E–G presents the distribution of complement proteins C3 and C4, along with anti-dsDNA. As expected, most measurements of C3 and C4 fall below the normal reference ranges (0.90–1.80 g/l for C3 and 0.10–0.40 g/l for C4), indicative of complement consumption common in SLE due to immune complex formation and inflammation. Fig. 2G highlights the elevation of anti-dsDNA levels above the normal upper limit of <100 IU/ml across the cohort.

**Table 1.** Baseline clinical and laboratory characteristics for 60 patients with systemic lupus erythematosus (SLE) and 60 with rheumatoid arthritis (RA)

SLE (N = 60)	Clinical variables
SLEDAI-2K^a^	6.0 (4.0, 8.0)
Organ involvement^b^	
Cardiovascular/Respiratory	5 (8.3%)
Constitutional	11 (18%)
Mucocutaneous	12 (20%)
Musculoskeletal	32 (53%)
ANA-positive patients^b^	
1:1280	11 (18%)
1:2560	21 (35%)
1:640	28 (47%)
C3 (g/L)^1^	0.83 (0.70, 0.98)
C4 (g/L)^1^	0.12 (0.10, 0.16)
Anti-dsDNA (IU/mL)^a^	188 (127, 237)
ENA-positive patients^b^	
Anti-Sm	56 (93%)
Anti-SSA	20 (33%)
Anti-SSB	7 (12%)
Anti-U1RNP	25 (42%)

RA (N=60)	Clinical variables

Initial articular involvement pattern^b^	
Monoarticular	17 (28%)
Oligoarticular	20 (33%)
Polyarticular	23 (38%)
RF-positive^b^	50 (83%)
Anti-CCP-positive^b^	60 (100%)
SvdH score^b^	
1	23 (38%)
2	17 (28%)
3	11 (18%)
4	9 (15%)

a Median (IQR).

b Counts (percentages).

For RA patients, Table 1 displays the patterns of initial articular involvement, and serological findings such as RF and anti-CCP positivity. This data is complemented in Fig. 2H that illustrates the distribution of Sharp/van der Heijde (SvdH) scores, reflecting varying degrees of joint damage: 23% of patients have a score of 1, indicating minimal joint damage suggestive of early or mild RA; 17% have a score of 2; 11% have a score of 3; and 9% have a score of 4, the latter two categories indicating more severe joint erosion and deformation. Although fewer patients fall into these higher score categories, their presence underscores the heterogeneity of disease progression within the cohort.

Fig. 2I depicts the patterns of initial articular involvement, showing that approximately one-third of patients have monoarticular involvement, one-third have oligoarticular involvement and one-third have polyarticular involvement. Fig. 2J indicates an 83% positivity rate for RF.

In the upper part of Table 2, we present demographic variables (age and gender) alongside plasma levels of SDC-1 and FLCs, specifically k-FLC, λ-FLC and the k/λ ratio, measured in the recruited RA and SLE patients. Data are compared with those measured in a group of 50 HD, using a non-parametric Kruskal–Wallis test. Table 2 highlights significant differences in age among HD patients (median age 45 years), SLE patients (median age 48 years) and RA patients (median age 56 years). No significant differences are observed regarding gender.

**Table 2.** Univariate analysis and multivariable linear regression

Univariate analysis				
Variable	HD, N = 50^a^	RA, N = 60^a^	SLE, N = 60^a^	*P*-value*^b^*

Age (years)	45 (38, 54)	56 (52, 65)	48 (41, 54)	<0.001
Gender				0.12
F	31 (63%)	48 (80%)	46 (77%)	
M	18 (37%)	12 (20%)	14 (23%)	
SDC-1 (ng/mL)	24 (17, 30)	126 (103, 158)	145 (116, 184)	<0.001
k-FLC (mg/L)	14 (12, 15)	63 (51, 76)	66 (50, 83)	<0.001
λ-FLC (mg/L)	12 (10, 13)	54 (43, 65)	61 (45, 74)	<0.001
k/λ	1.16 (1.04, 1.31)	1.22 (1.05, 1.34)	1.13 (1.06, 1.27)	0.4

Multivariable linear regression			
	Model 1	Model 2	Model 3
Independent/Dependent variable	Syndecan-1 (ng/ml)	k-FLC (mg/dl)	λ-FLC (mg/dl)

Intercept	25.5***	14.2***	12.0***
Centred age	0.20	0.047	−0.05
RA	113.5***	53.9***	46.0***
SLE	127.15***	55.1***	50.2***
*P*-value	<2.2·10^-16^	<2.2·10^-16^	<2.2·10^-16^
Adjusted $R^{2}$	0.61	0.53	0.48

Univariate analysis: we show demographic characteristics and plasma biomarker levels in healthy donors (HD), rheumatoid arthritis (RA) and systemic lupus erythematosus (SLE) patients. Median ages were 45 years (HD), 56 years (RA) and 48 years (SLE). Plasma levels of syndecan-1 (SDC-1) and specific free light chain (FLC) subtypes, namely k-FLC and λ-FLC, along with their k/λ ratio, were measured. Multivariable linear regression: we display multiple linear regression models with syndecan-1 (Model 1), k-FLC (Model 2) and λ-FLC (Model 3), as functions of centred age (obtained by subtracting the mean age of recruited subjects from each patient’s age), considering the presence of a diagnosis of RA or SLE. The regressions are performed using HD as a reference.

a Median (IQR); *n* (%).

b Kruskal–Wallis rank sum test; Pearson's χ^2^d test.

* *P* < 0.05,

** *P* < 0.01,

*** *P* < 0.001 and

**** *P* < 0.0001.

Statistically significant differences are observed in the levels of SDC-1, k-FLC and λ-FLC, which are higher in the pathological groups (RA and SLE) compared with HDs. Conversely, no statistically significant difference is observed in the k/λ ratio among the three groups.

**Figure 3.**
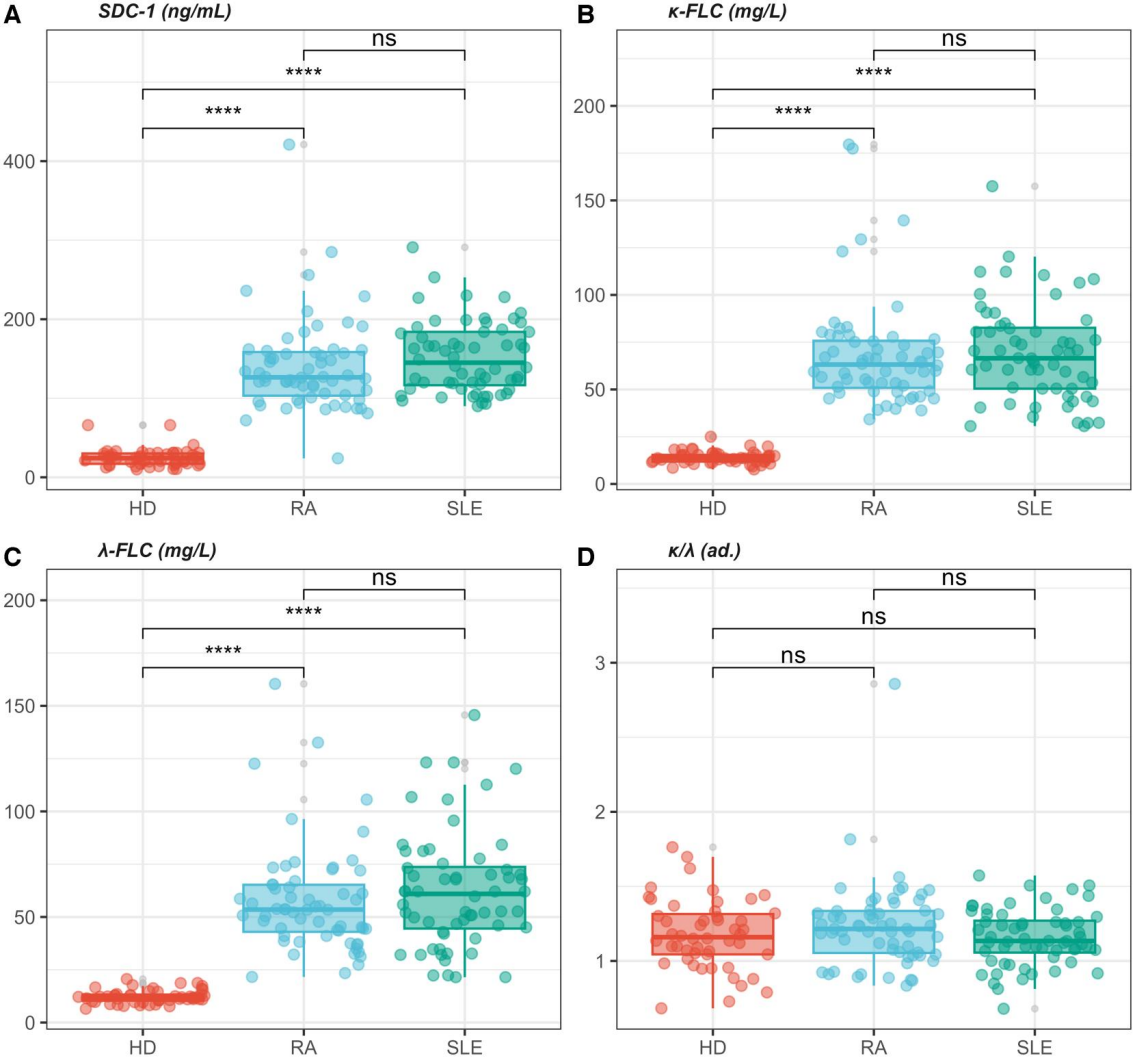
Box plot analysis illustrating levels of syndecan-1 and free light chains. SDC-1 (**A**), k-FLC (**B**), λ-FLC (**C**) and their ratio (**D**). Post-hoc analysis, using a Bonferroni-corrected Wilcoxon Mann–Whitney *U* test, highlights significant differences between the pathological groups and healthy controls, while no significant differences are observed between RA and SLE patients. Significance levels are indicated by asterisks: *P < 0.05, **P < 0.01, ***P < 0.001 and ****P < 0.0001

A box plot analysis of the discussed variables is shown in Fig. 3, reporting the levels of SDC-1 (Fig. 3A), k-FLC (Fig. 3B), λ-FLC (Fig. 3C) and their ratio (Fig. 3D). The post-hoc analysis conducted with a Bonferroni-corrected Wilcoxon Mann–Whitney *U* test points out that differences are attributable to a distinction between the two pathological groups and HDs. In contrast, no significant differences are observed between RA and SLE patients, despite the latter showing slightly higher values of all the investigated markers.

The potential confounding effect of age on this univariate analysis is assessed in the lower half of Table 2, where we show the outcomes of three bivariate linear regression models. In these models, age and group membership (HD, SLE, RA) are used as explanatory variables, while SDC-1 (model 1), k-FLC (model 2) and λ-FLC (model 3) are employed as dependent variables. To achieve this, ages were centred on the mean age of the recruited subjects. The models, conducted using HDs as reference, are all extremely significant with a *P*-value $2.2\times 10^{-16}$ and with a substantial adjusted-R^2^ of ∼50% of the variance or greater. Specifically, the regression indicates that the coefficients related to the patient’s clinical status (SLE or RA) are highly statistically significant, while age is not significant. This suggests a small confounding effect due to age in the previously discussed univariate results. The coefficients for RA and SLE offer an age-adjusted estimate of variations in the levels of the investigated markers compared with levels measured in a healthy donor with the mean age. Accordingly, the intercept provides an estimate of the levels of the investigated parameter in a healthy subject with an age equal to the mean age of the recruited subjects. The regression enables us to quantify an average significant increase of 113.5 (127.15) ng/ml, 53.9 (55.1) mg/dl and 46.0 (50.2) mg/dl in SDC-1, k-FLC and λ-FLC in subjects with RA (SLE) compared with HDs.

**Figure 4.**
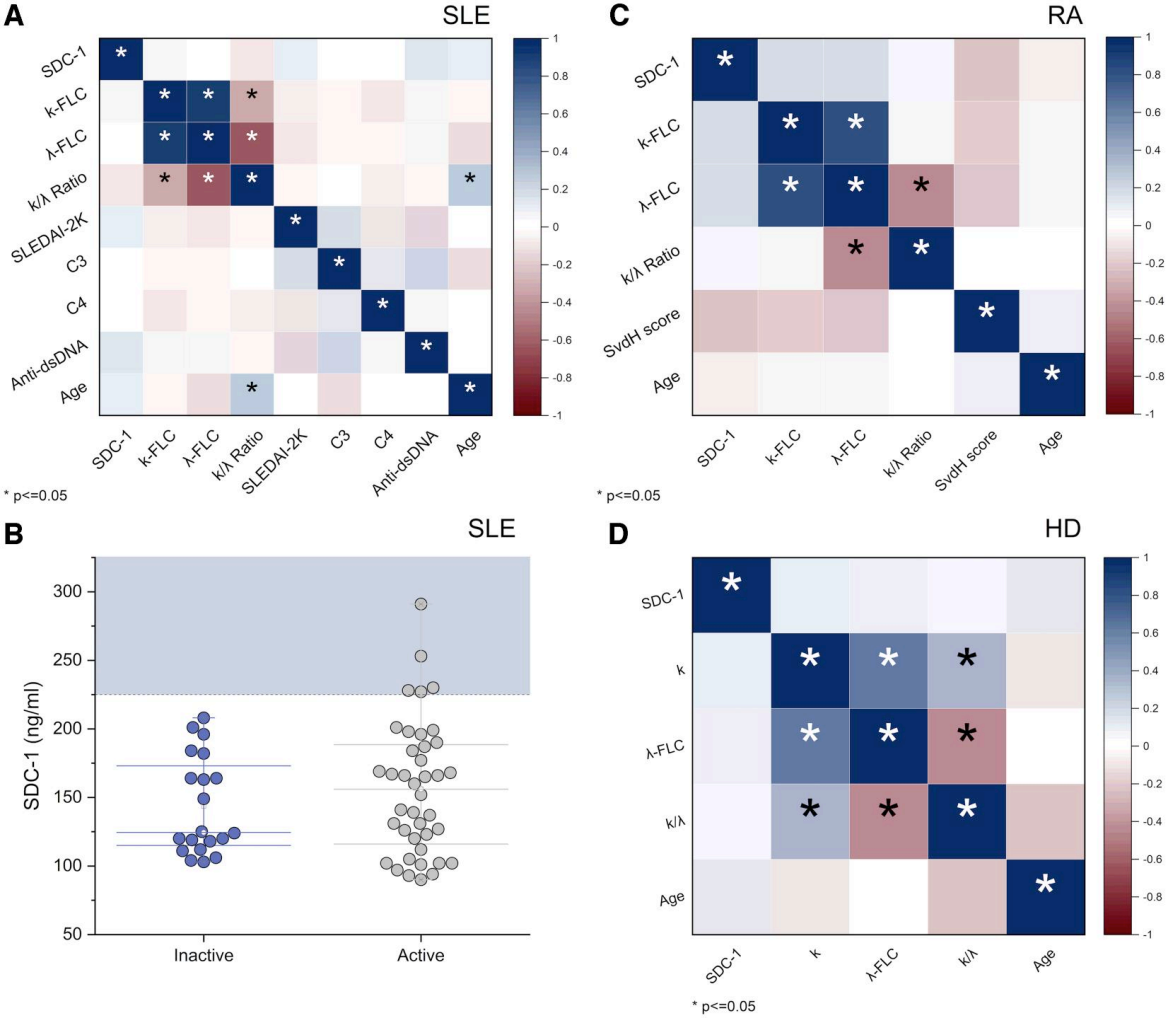
Correlational analysis. Correlations among demographic parameters (age), serum levels of syndecan-1, free light chains (κ, λ and their ratio) and disease activity indexes, when available. Correlations are evaluated using the Spearman correlation coefficient and are arranged in correlation matrices. (**A**) Correlational data for SLE patients; (**B**) syndecan-1 levels in SLE patients with active disease (SLEDAI-2K >6) *vs* inactive disease (SLEDAI-2K ≤6); in **C** and **D** we report correlational data for RA patients and healthy donors, respectively. A colour code is used for the correlation matrices: strong positive correlations are represented in dark blue, and strong negative correlations are indicated in dark red. Significant correlations are marked with an asterisk (*), with significance set at *P* < 0.05

In Fig. 4, we present an analysis of the correlations between the investigated markers, namely FLCs and SDC-1, considering also their relation to disease activity. The correlations are quantified using Spearman’s coefficients, displayed in correlation maps. Fig. 4A focuses on SLE patients, showing expected significant correlations among different FLCs, but not between FLCs and SDC-1. A significant positive correlation is observed between the k/λ ratio and the age of the subjects. Interestingly, the analysis reveals no statistically significant correlation between FLCs and SDC-1 levels and indicators of disease activity, including SLEDAI-2K, C3 and C4.

Fig. 4B further examines the association between SDC-1 levels and disease activity through a box plot analysis. For this purpose, data were stratified into active and non-active disease groups based on the SLEDAI-2K score being above 6 or not. Although no statistically significant differences are noted, as confirmed by a Wilcoxon Mann–Whitney test, it is observed that SDC-1 levels above 225 mg/ml are present only in cases of active disease.

The correlation analysis is also presented in Fig. 4C for RA patients and compared in Fig. 4D for HDs. Similar to SLE patients, there is a correlation among different FLCs but not between these and SDC-1 levels. Unlike the SLE group, no significant correlation with age is noted in RA patients. Furthermore, like SLE patients, no statistically significant correlation is observed with activity indices, measured via the SvDH score.

## Discussion

Here, we investigate the role of SDC-1 and FLCs as biomarkers for SARDs, using SLE and RA as model systems. SDC-1, a member of the transmembrane proteoglycans, plays a significant role in autoimmune diseases, with its complex involvement in both inflammation and tissue repair [19, 24, 26, 29, 40–42]. Its ability to interact with a many ligands is associated with its ectodomain encompassing chondroitin and heparan sulphate chains (Fig. 1) [24]. This feature facilitates engagement with various cellular and extracellular molecules, including growth factors, adhesion receptors, cytokines, chemokines, proteinases and other ECM components. Its predominant expression on epithelial and endothelial cells, as well as on plasma cells, coupled with its versatility in cellular signalling, positions SDC-1 as a key biological regulator. Despite this pivotal role, the mechanisms behind its action in inflammation and autoimmunity are still unclear. This complexity arises partly from SDC-1’s multifaceted behaviour, as it is capable of exhibiting both anti-inflammatory and pro-inflammatory properties depending on the tissue context, the investigated disease and whether its soluble or membrane-bound form is involved [41]. In fact, SDC-1 can be released from cells through the process of shedding in response to, for example, cellular stress, leucocyte-derived protease accumulation and growth factor release, helping to maintain a proteolytic and growth factor balance, as well as mediating inflammation [43]. Accordingly, the release of SDC-1 has been observed in various pathological conditions, suggesting its role as a potential biomarker [44].

Here, we observed elevated levels of soluble SDC-1 in RA patients compared with controls, with an age- and gender-adjusted difference of ∼114 ng/ml. Given the SDC-1 dual function, both pro-inflammatory and anti-inflammatory, it would be interesting for future studies to determine which of these roles predominates in this context. Regarding the latter, Salminen-Mankonen *et al.* demonstrated a SDC-1 increase in chondrocytes from cartilage damaged in the early stages of osteoarthritis, suggesting a potential involvement in the mechanisms that repair damaged joints [45]. Jurjus *et al.* found that the absence of SDC-1 in a collagen II-induced arthritis mouse model led to more severe symptoms [19]. Consistently, it has been shown that anti-rheumatic drugs decrease the serum level of SDC-1, which might reflect a decrease in shedding [30]. To limit this alteration and avoid bias in level estimates, we recruited patients who had discontinued their immunosuppressant for 3–6 months. At variance with our results, a different scenario is depicted by Kim *et al.* describing that SDC-1 levels were comparable between RA patients and controls [26]. This likely arises from specific differences in the clinical baseline of the recruited patients. In our case, the reported values pertain to patients with untreated RA in various states of disease activity, as indicated in Fig. 2, where we observe that 33% of patients have a SvdH score between 3 and 4. This variability in disease activity might account for the elevated SDC-1 values observed in our cohort.

Notably, SDC-4, more often that SDC-1, is associated with RA [46], a fact that might be related to the tendency of syndecans to influence the expression of each other [41].

We also report elevated levels of SDC-1 in SLE patients compared with controls. This elevation supports the role of SDC-1 in the pathogenesis of SLE, consistent with both clinical literature and experimental findings [29]. Specifically, Minowa *et al.* demonstrated that serum SDC-1 levels were higher in SLE patients with active disease compared with those with inactive disease [47]. These findings are corroborated by Kim *et al.* who observed that serum SDC-1 levels were higher in SLE patients with nephritis compared with those without [26]. The consistency across various studies, including our own, further supports the utility of serum syndecan-1 as an SLE biomarker.

At variance with other studies, our correlational analysis does not reveal a significant correlation between disease activity levels (SLEDAI 2K for SLE, and SvdH for RA). This discrepancy might be attributed to the specific range of scores spanned by our patients. Alternatively, the difference could stem from the statistical analysis employed. In this regard, some studies in the literature use parametric methods (Pearson coefficient or linear regression) which are susceptible to outliers; this is common in the statistical distributions of inflammatory biomarkers, particularly in pathological subjects. This can lead to spurious correlations, an issue generally better managed with non-parametric methods, such as those used here. This issue is intriguing and warrants further methodological exploration in subsequent studies.

Interestingly, the literature indicates some uncertainty about whether the increase in soluble SDC-1 levels in SLE patients is only indicative of renal activity or if it also plays a role in glomerular pathology [29]. Our results, which include data from SLE patients without any sign of renal damage, lean towards the latter.

Aside from autoimmune diseases, elevated levels of SDC-1 in serum have been associated with conditions that involve the expansion and activation of plasma cells, such as multiple myeloma [23]. Similarly, it has been suggested that high serum levels of SDC-1 in SLE could also be linked to an increased activation of plasma cells. Interestingly, the literature indicates a complex interplay between serum SDC-1 and activated plasma cells, which is likely bidirectional in nature. On the one hand, released SDC-1 into plasma remains active, affecting the behaviour of plasma cells. On the other hand, the serum levels of SDC-1 could be augmented as a consequence of plasma cell activation [47]. Activation of plasma cells, in turn, contributes to the pathogenesis of SLE and RA by producing pathogenic autoantibodies and, consequently, an excess of k- and λ-FLCs [16]. Indeed, aside from high levels of SDC-1, we also find elevated levels of k- and λ-FLCs in patients compared with controls. Notably, as these are not lymphoproliferative disorders, the k/λ ratio is not altered.

While the activation of plasma cells likely contributes to the elevated levels of all markers investigated in this study, we do not observe a direct correlation between FLCs and SDC-1 when examining the groups separately. The absence of a direct correlation might be due to different mechanisms responsible for their release and deserves more in-depth studies in future works [15, 16, 22, 23].

Limitations are correlated with patient recruitment, particularly the sample size. The findings of this study need confirmation and extension in a larger cohort to identify more subtle differences, including disease-specific alterations between SLE and RA. Additionally, we exclusively included subjects who had suspended immune-suppressants to ensure that the marker levels were not markedly affected by pharmacological treatment. Therefore, our study lacks comparison groups, such as patients at the time of diagnosis and those undergoing post-therapy evaluation. This limits our understanding of the dynamics of SDC-1 levels across different disease and treatment stages, preventing us from drawing comprehensive conclusions about the role of SDC-1 in the disease’s progression and response to therapy.

## Data Availability

The data presented in this study are available upon reasonable request from the corresponding author.
